# Genetic variants in interferon-λ 4 influences HCV clearance in Chinese Han population

**DOI:** 10.1038/srep42408

**Published:** 2017-02-10

**Authors:** Peng Huang, Yinan Yao, Ming Yue, Ting Tian, Hongbo Chen, Mingzhu Chen, Jie Wang, Yun Zhang, Rongbin Yu

**Affiliations:** 1 Department of Epidemiology and Biostatistics, School of Public Health, Nanjing Medical University, Nanjing 211166, China; 2 Department of Infectious Diseases, the People’s Hospital of Jiangsu Province, Nanjing 210029, China; 3 Department of Infectious Diseases, the Jurong Peoples’ Hospital, Jurong 212400, China; 4 School of Nursing, Nanjing Medical University, Nanjing 211166, China; 5 Huadong Research Institute for Medicine and Biotechnics, No. 293 Zhongshan East Road, Nanjing 210002, Jiangsu, China

## Abstract

Recent many studies indicated a novel dinucleotide variant in ss469415590 (TT vs. ΔG) of interferon-λ 4 (*IFNL4*) gene strongly associated with hepatitis C virus clearance. To evaluate the impact and clinical usefulness of *IFNL4* ss469415590 genotype on predicting both spontaneous HCV clearance and response to therapy in Chinese population, we genotyped 795 chronic HCV carriers, 460 subjects with HCV natural clearance and 362 patients with pegylated interferon-α and ribavirin (PEG IFN-α/RBV) treatment. *IFNL4* ss469415590 variant genotypes significantly decreased host HCV clearance, both spontaneous (dominant model: OR = 0.50, 95% CI = 0.36–0.71) and IFN-α induced (dominant model: OR = 0.32, 95% CI = 0.18–0.56). Multivariate stepwise analysis indicated that ss469415590, rs12979860, the level of baseline HCV RNA and platelet were as independent predictors for sustained virological response (SVR). But the area under the ROC curve (AUC) was only 0.58 for ss469415590, and it was elevated to 0.71 by adding rs12979860, baseline HCV RNA and platelet in the prediction model of SVR. Therefore, these findings underscore that although genetic factors of host and pathogen were commonly important during HCV clearance, ss469415590 may be also a strongly predictive marker in the Chinese population.

The hepatitis C virus (HCV) prevalence was about 2.3% all over the world, with 170 million HCV-infected individuals and 29 million in China^1^,^2^. Chronic hepatitis C (CHC) is one of the most common chronic blood-borne infection, and about 30% patients persist infections will progress chronic liver disease, including cirrhosis and hepatocellular carcinoma (HCC)^3^. About 50% of CHC patients infected with HCV genotype 1 could achieve sustained virological response (SVR) under the therapy of pegylated IFN-α and ribavirin (PEG IFN-α/RBV), while the rate may increase to 70–90% for those infected with genotype 2/3^4^.

The eradication of HCV, both spontaneous clearance and IFN-α induced, is influenced by a complex interaction of environmental factors, viral characteristic and genetic background. Genome-wide association studies (GWAS) have identified susceptibility loci at interleukin-28B (*IL28B*) region^5^,^6^. Recently, Prokunina-Olsson *et al*. and Bibert *et al*. has found a dinucleotide variant in ss469415590 (TT vs. ΔG), which located in upstream of *IL28B* and was in strong linkage disequilibrium (LD) with rs12979860^7^,^8^. This novel SNP leads a frame shift mutation leading to product a novel gene, interferon-λ 4 (*IFNL4*) which 40.8% amino acid sequence was in consistent with *IFNL3*. Interestingly, the expression of hepatic interferon stimulated genes (ISGs) was triggered by *IFNL4* not *IL28B*, suggesting that *IFNL4* play a key role in responding to exogenous interferon^9^,^10^. Moreover, many researchers confirmed that HCV clearance and induction of IL28B and CXCL10 mRNA depended on ss469415590 genotype rather than rs12979860 and ss469415590 may be the truly functional variant^11^,^12^.

To further test the association of *IFNL4* ss469415590 variants with spontaneous HCV clearance and response to treatment, we genotyped IFNL4 ss469415590 and IL28B rs12979860 in 795 chronic HCV carriers, and 460 subjects with HCV natural clearance and 362 patients with PEG IFN-α/RBV treatment in Chinese Han populations.

## Results

### Participant characteristics

Participant profiles were shown in Table 1. Among 795 persistent HCV carriers and 460 subjects with spontaneous clearance, elder, female and subject infected with viral genotype 1 were significantly more likely to be chronic infection (*P* < 0.05 for both comparisons). And patients with spontaneous HCV clearance had normal aspartate aminotransferase (AST) and alanine aminotransferase (ALT) than persistent HCV cases (*P* < 0.001 for AST and *P* = 0.015 for ALT). In this study, 362 chronic infection patients treated with PEG IFN-α/RBV therapy were recruited from a population of former paid-blood donors, which the viral genotype of these CHC were all genotype 1. 65.7% patients achieved SVR. There were more patients with abnormal AST, higher baseline HCV RNA, and lower platelet among none sustained virological response (N-SVR) group (*P* = 0.038, <0.01, 0.039, respectively).

The observed genotype frequencies for the two SNPs in subjects with spontaneous HCV clearance were all in Hardy-Weinberg equilibrium (HWE) (*P* > 0.05). But the allele frequencies of rs12979860 were not in accordance with the predicted HWE in patients who attained SVR (*P* < 0.001). The LD was moderate between rs12979860 and ss469415590 both for spontaneous resolver (r^2^ = 0.62) and for patients who attained SVR (r^2^ = 0.79).

### ss469415590 variants and HCV spontaneous clearance

*IFNL4* ss469415590 genotype had a significant impact on spontaneous HCV clearance. Clearance rate was higher in patients carrying the beneficial TT/TT genotype (39.4%) than in patients with TT/ΔG (23.8%) or ΔG/ΔG (18.7%) (*P* < 0.001). Meanwhile, *IL28B* rs12979860 variants were also negatively related with viral clearance and clearance rate was 38.9%, 27.6% and 17.2% for CC, CT and TT genotype (*P* < 0.001). After adjusting for age, gender, and viral genotype, logistic regression analyses showed that *IFNL4* ss469415590 and *IL28B* rs12979860 variant genotypes significantly decreased the ability of host HCV clearance (dominant model: OR = 0.50, 95% CI = 0.36–0.71 for ss469415590; OR = 0.59, 95% CI = 0.42–0.83 for rs12979860) (Table 2). This effect of ss469415590 and rs12979860 on HCV clearance remained in existence after being conditioned on each other (*P* = 0.005 for ss469415590 and *P* = 0.016 for rs12979860) and therefore they was included in further combined analyses by adding up the number of unfavorable alleles of the independent SNPs ss469415590-ΔG and rs12979860-T. The result showed that subjects carrying three to four unfavorable alleles had a 90% decrease in clearance in comparison with those without unfavorable allele (OR = 0.10, 95% CI = 0.02–0.42) (Table 3).

### ss469415590 variants and response to treatment

In 361 HCV genotype 1 patients, SVR was strongly associated with *IFNL4* ss469415590 genotype: 70.0% of patients with ss469415590 TT/TT had SVR, whereas only 43.6% of patients with TT/ΔG and 16.7% of ΔG/ΔG achieved SVR (*P* < 0.001). Subjects with *IL28B* rs12979860 variant TT were also fewer to achieve SVR (Table 2). Multivariate stepwise analysis indicated that these two SNPs, the level of baseline HCV RNA and platelet were as independent predictors for SVR (Table 4). After adjustment with age, gender, baseline HCV RNA and platelet, multivariate logistic regression showed that the OR of ss469415590 and rs12979860 was 0.32 and 0.34 in dominant model respectively, compared with patients with favorable genotypes (Table). The combined effect of unfavorable alleles on SVR indicated that carrying two unfavorable alleles offered the highest risk effect (OR = 0.13, 95% CI = 0.07–0.25), as showed in Table 3.

**Figure 1:**
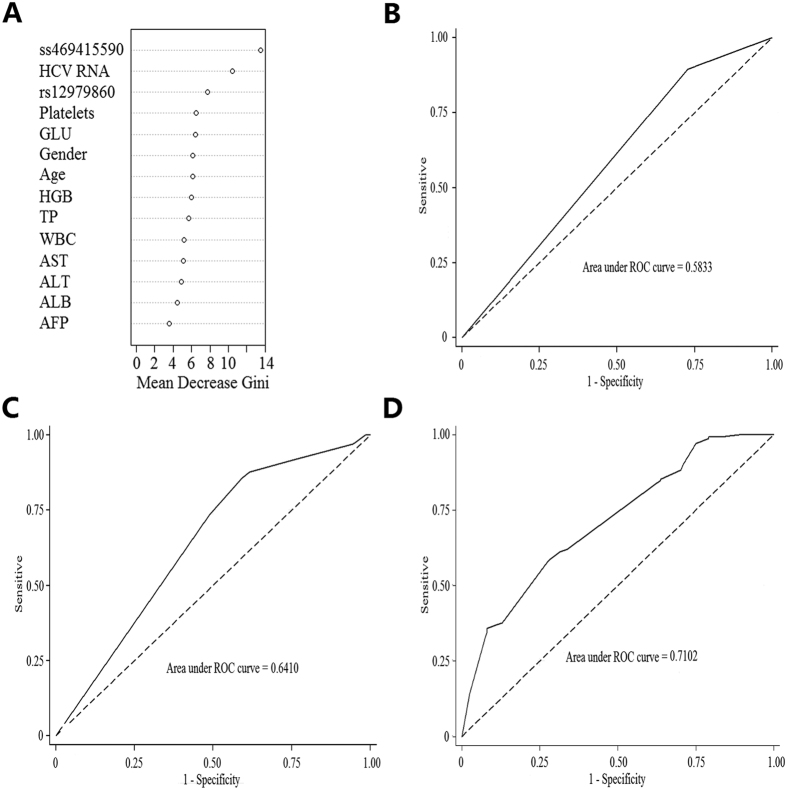
Predictors of HCV treatment response. (**A**) Importance blots of all variables in 362 patients. (**B**) ROC curve for prediction of treatment response using the *IFNL4* ss469415590. (**C**) ROC curve for prediction of treatment response using the combination of *IFNL4* ss469415590 and *IL28B* rs12979860. (**D**) ROC curve with the combination of *IFNL4* ss469415590, *IL28B* rs12979860, baseline HCV RNA and platelet to predict HCV treatment response.

We used decision tree ensembles in the form of a random forest classifier to quantify the relative predictive power of ss469415590, rs12979860, selected demographic characteristics, and clinical features. As a result of this multivariate analysis, the predictive power of a variable was expressed as the Gini. The Fig. 1A showed that the first four stronger factors were ss469415590, baseline HCV RNA, rs12979860 and platelet. The AUC was 0.58 for ss469415590, and was 0.64 for the set of ss469415590 and rs12979860 (Fig. 1B,C). But the AUC was elevated to 0.71 by adding baseline HCV RNA and platelet in the prediction model, suggesting that genetic factors of host and pathogen were commonly important to resolution of virus during treatment (Fig. 1D).

Meanwhile ss469415590-ΔG variant (dominant model: OR = 0.26, 95% CI = 0.12–0.54 for RVR; OR = 0.11, 95% CI = 0.06–0.22 for EVR) and rs12979860-T variant (dominant model: OR = 0.37, 95% CI = 0.23–0.62 for RVR; OR = 0.46, 95% CI = 0.26–0.81 for EVR) were also negatively related with RVR and EVR (Supplemental Table 2).

### ss469415590 variants and HCV time-dependent clearance

**Figure 2:**
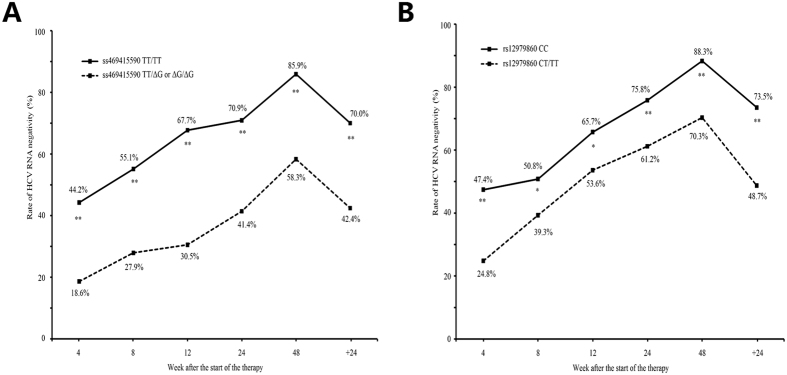
Effect of *IFNL4* and *IL28B* variants on time-dependent clearance of HCV. (**A**) The rate of undetectable HCV RNA stratified by *IFNL4* ss469415590 alleles. (**B**) The rate of undetectable HCV RNA stratified by *IL28B* rs12979860 alleles. *P < 0.05, **P < 0.01.

Patients were stratified according to their *IFNL4* ss469415590 and *IL28B* rs12979860 allele type, and the rate of undetectable HCV-RNA at 4, 8, 12, 24, 48 weeks after the start of therapy and 24 weeks after cessation of treatment were analyzed (Fig. 2A,B). The rate of undetectable HCV RNA was significantly higher in patients with the ss469415590 TT and rs12979860 CC genotype than ss469415590 TT/ΔG or ΔG/ΔG and rs12979860 CT/TT genotype. Especially, the difference was most significant when stratified by ss469415590 genotype: 44.2% vs. 18.6% for 4 weeks, 67.7% vs. 30.5% for 12 weeks and 85.9% vs. 58.3% for 48 weeks.

### Meta-analysis

**Figure 3:**
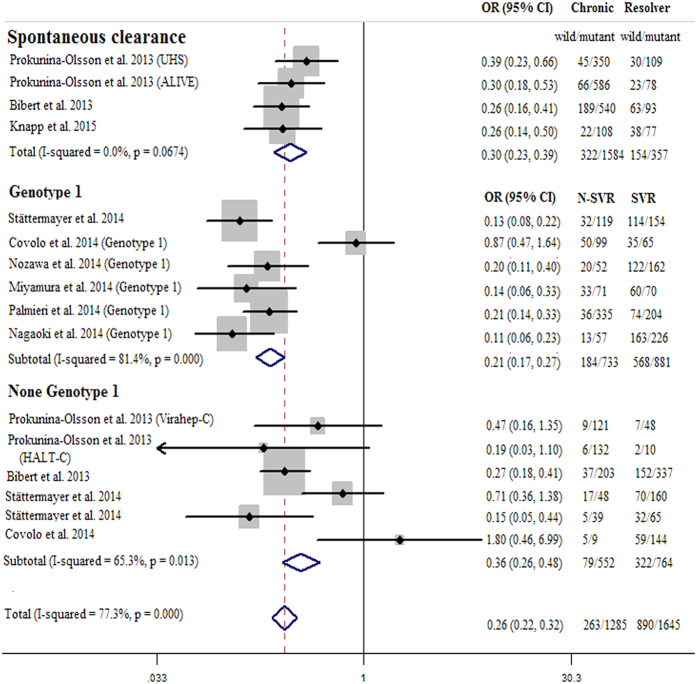
Meta-analysis of the *IFNL4* ss469415590 on HCV clearance in dominant genetic model. The position of each symbol indicates the odds ratio (OR) for each included study; the horizontal lines indicate 95% confidence intervals (95%CIs). The vertical dashed lines represent the overall OR of all included studies. Arrows indicate 95%CIs that exceed the x-axis scale.

To further study the impact of *IFNL4* ss469415590 on HCV clearance, a meta-analysis was performed by the published data, including four data for HCV spontaneous clearance and 12 data for HCV SVR (Supplemental Table 3)^7^,^8^,^11^,^12^,^13^,^14^,^15^,^16^,^17^. Figure 3 indicated significant association between *IFNL4* ss469415590 variant and decreased ability of HCV clearance (pooled OR = 0.30, 95% CI = 0.23–0.39 for HCV spontaneous clearance; pooled OR = 0.26, 95% CI = 0.22–0.32 for HCV SVR in dominant model). Subgroup analysis showed that the estimated OR was 0.21 (95% CI = 0.17–0.27) in HCV genotype 1 and 0.36 (95% CI = 0.26–0.48) in other genotypes. These results indicated that patients with ss469415590 variant types may have a lower ability of HCV clearance, especially in viral genotype 1.

## Discussion

In this study, we investigated the associations between *IFNL4* ss469415590 and risk of HCV clearance and this is the first study that validated the observations of Prokunina-Olsson *et al*. and of Bibert *et al*. on the prediction of the eradication of HCV, both spontaneous and IFN-α induced, in a large cohort of Chinese Han populations^7^,^8^. The main finding was that the predictive value of *IFNL4* ss469415590 was almost identical to that of *IL28B* rs12979860, which were all consistent with previous studies in other races^12^,^13^,^14^. But, unexpectedly, the linkage disequilibrium among these two SNPs in our study was only moderate, especially in spontaneous resolver (r^2^ = 0.62). Previous studies have reported that ss469415590 and rs12979860 were in strong linkage disequilibrium in chronic hepatitis C virus type 1 or 4 infection in Caucasians^12^,^18^. Both studies were conducted to predict the treatment response of peginterferon alpha and ribavirin. However, the moderate linkage disequilibrium in our study was performed in spontaneous resolver. Besides, the linkage disequilibrium between ss469415590 and rs12979860 in Chinese Han population may be not as strong as in Caucasians.

We performed a mini meta-analysis with a dominant genetic model by pooling the published data to study the effect of *IFNL4* ss469415590 on HCV clearance. The pooled ORs were lower than these in our current research for both spontaneous clearance and IFN-α induced eradication, suggesting the negative effectiveness of *IFNL4* ss469415590 variant was stronger in the meta-analysis. The mainly reason may be the discrepancy of genetic background in different races. The frequency of ss469415590-TT/ΔG or ΔG/ΔG was about 12.2% in Chinese patients with HCV spontaneous clearance of our study, while that was about 69.9% in non-Chinese patients of the meta-analysis. This extreme variation also was found in SVR patients infected with genotype 1 (10.5% vs. 60.8%). But this discrepancy of frequency of ss469415590-TT/ΔG or ΔG/ΔG in SVR patients infected with genotype 1 was reduced among our study and three Japanese studies of the meta-analysis (10.5% vs. 24.7%).

Although GWAS defined the strong association between *IL28B* SNPs and viral eradication in hepatitis C, the exact mechanism or function of *IL28B* has yet to be testified, and even some paradoxes existed in various researches. On one hand, based on different *IL28B* alleles, the level of expression of *IL28B* was dissimilar between in peripheral blood mononuclear cells (PBMCs) and in liver tissue. Many studies identified that the level of expression of *IL28B* was lower in PBMCs from subjects with unfavorable *IL28B* variants in comparison with patients carrying the wild-type gene. However, this findings was fewer confirmed in liver tissue^6^,^19^. On the other hand, the causal impact of IL28A which was a homology with IL28B on HCV clearance was hardly recognized, suggesting that IL28B may not be the true explanation of HCV clearance^9^. Recently, several researches focused on the function of *IFNL4* which may provide the causal mechanism of HCV clearance. First of all, as is known, patients with a high induction of ISG in liver tissue would achieve a poor response to IFN-α. And ISGs upon transient expression in hepatocyte was induced by *IFNL4* gene transfection^20^. In addition, the study of Bibert *et al*. confirmed that the level of IL28B and CXCL10 expression in poly(I:C)-stimulated PBMCs was strongly depended on ss469415590 genotypes but not on rs12979860^8^. Covolo *et al*. validated the observations that circulating level of CXCL10 was significantly higher in patients with ss469415590 mutant alleles^12^.

Although *IFNL4* ss469415590 is strongly associated with HCV clearance, it is uncertain that these genetic markers will be still needed if the upcoming IFN-free therapy will become available. We think this conjecture is not well founded. Firstly, many new treatments such as Daclatasvir and Sofosbuvir were still in clinical trials and such high payment lead it will not be available for the foreseeable future in developing countries^21^. Secondly, with significantly high SVR rates and safety, these IFN-free therapies were researched only in recent years and still needed a long follow-up time to assess the safety and efficacy^22^. Thus, interferon-based therapies will be used for many years in most countries. Finally, in the recent paper on the triple therapy with pegylated IFN-α2b, RBV and telaprevir, 176 patients with *IFNL4* ss469415590 TT/TT had better response rates than those carrying TT/ΔG or ΔG/ΔG alleles, suggesting genetics may still play a role for new therapy^16^.

In this study the environment factors (baseline viral load and platelet) as well as genetic factors independently contribute to the response to treatment. The predict value of ss469415590 for SVR was only moderate (AUC = 0.58) and almost 30% patients carrying with beneficial IFNL4 TT/TT genotype did not achieve SVR. These results were confirmed by some recent findings, and reflected a complex genotype -environment interaction for predicting HCV clearance.

Our study had numerous strengths. First of all, enough statistical power was guaranteed with a relatively large sample size in this study, and it is the first study demonstrating that *IFNL4* ss469415590 variants influence HCV clearance in Chinese Han population, which validated the effect of the novel marker in non-Caucasians. Moreover, patients with treatment came from parts of HCV persistent carriers who were all infected with HCV by blood donation, which may have reduced potential selection bias. Finally, it is rare to study the association between *IFNL4* ss469415590 and HCV clearance, both spontaneous and IFN-α induced in one study. However, little is known regarding the biological mechanism of the significant SNP in the clearance of HCV. Therefore, validations with functional characterizations are warranted.

In conclusion, our study validated that *IFNL4* ss469415590 was also strongly associated with HCV clearance in Chinese Han population.

## Methods

### Ethical statement

This study was approved by the medical ethics committee of Nanjing Medical University. All subjects provided informed consent to participate in the study. The experiment was carried out according to the Declaration of Helsinki.

### Participants

1255 participants for studying spontaneous HCV clearance were recruited from the Nanjing compulsory detoxification center (Nanjing, China) during May and Dec 2006, nine hospital hemodialysis centers in southern China during Oct 2008 and Jan 2010, and a population of former paid-blood donors (Zhenjiang, China) from April 2010 to January 2013. The information about these participants had been described in our previous studies^23^,^24^. 362 treatment-naïve CHC patients were recruited from Jurong People’s Hospital (Jurong, China) from Jan 2011 to Oct 2013, which aimed to evaluate the factors that impacted on response to anti-viral therapy. Eligibility criteria for therapy included age between 18 and 70 years, detectable HCV RNA in serum over a span of more than 6 months of treatment initiation, negative for hepatitis B infection, without other types of liver diseases such as alcoholic diseases, autoimmune liver diseases or metabolic liver diseases.

Each participant was interviewed by a structured questionnaire to collect information on demographic data and environmental exposure history. After interview, an approximately 5 mL venous blood sample was collected from each participant. The serum and peripheral blood mononuclear cells were separated and stored at −70 °C until assay.

### Treatment therapy and outcome

Patients received weekly injections of pegylated IFN-α2a (180 g) and ribavirin (RBV) was administered orally for 48 weeks. The amount of ribavirin was adjusted based on body weight (600 mg for <60 kg, 800 mg for 60–80 kg, 1000 mg for >80 kg).

According to the manufacturers’ instructions, HCV RNA was quantified in all patients at baseline and after 4, 12, 24 and 48 weeks of treatment and 24 weeks after cessation of treatment by Cobas Amplicor HCV Monitor Test, v2.0 (Roche, Basel, Switzerland). In this study, rapid virological response (RVR) was defined as undetectable HCVRNA at 4 weeks during therapy. Early virological response (EVR) was defined as ≥2 log reduction in HCV RNA level compared to baseline HCV RNA level or undetectable HCVRNA at 12 weeks during treatment. SVR was defined as HCV RNA negative 24 weeks after treatment-free follow-up.

### SNP genotyping

DNA extraction was with protease K digestion and phenol-chloroform purification, which was described previously^25^. Genotyping was performed by the TaqMan allelic discrimination assay on ABI PRISM 7900HT Sequence Detection system (Applied Biosystems, San Diego, CA, USA). The information on primers and probes are shown in Supporting Table 1. Two blank controls and 5 repeated samples were assigned in each genotyping assay, and a 100% concordant was achieved. The *IL28B* rs12979860 allele C and *IFNL4* ss469415590 alleles TT were defined as wild-type alleles, whereas the T and ΔG as mutant alleles respectively.

### Statistical analysis

Differences in the general demographic characteristics were calculated by the Student *t* test and the chi-square (χ^2^) test. The associations of SNPs with HCV spontaneous clearance, RVR, EVR and SVR were estimated by the odds ratios (ORs) and 95% confidence intervals (CIs) using multivariate logistic regression analysis. Age, gender and viral genotype were adjusted for HCV spontaneous clearance, while, age, gender, baseline HCV RNA and platelet were adjusted for RVR, EVR and SVR during regression analysis. A stepwise forward procedure was used for selecting the final logistic regression model for predicting outcomes of HCV treatment. The trend analysis was assessed with Cochran-Armitage trend test.

Random forest, an advanced tree classifier, was used to evaluate the importance of each variable and to improve the performance of classification. The Gini or information entropy was the standard to classify the partner node into two child nodes. In our study, the area under the curve (AUC) and the plot of variables importance indicated the result of this method^26^.

All the statistical analyses were carried out by STATA 12.0 software (StataCorp LP, College Station, TX, USA), and *P* < 0.05 in a two-sided test was considered as statistical significance.

## Additional Information

**How to cite this article**: Huang, P. *et al*. Genetic variants in interferon-λ 4 influences HCV clearance in Chinese Han population. *Sci. Rep.* **7**, 42408; doi: 10.1038/srep42408 (2017).

**Publisher's note:** Springer Nature remains neutral with regard to jurisdictional claims in published maps and institutional affiliations.

## Supplementary Material

Supplementary Information

## Figures and Tables

**Table 1:** Patient characteristics.

Variables	Spontaneous clearance (n = 1255)			Response to treatment (n = 362)		
	Chronic (n = 795) N(%)	Resolver (n = 460) N(%)	*P* value	N-SVR (n = 125) N(%)	SVR (n = 237) N(%)	*P* value
Age, year^a^	52.44 ± 12.30	47.05 ± 14.04	<0.001	53.91 ± 8.06	53.20 ± 8.81	0.456
Gender			0.017			0.883
Male	301 (37.9)	206 (44.8)		30 (24.0)	59 (24.9)	
Female	494 (62.1)	254 (55.2)		95 (76.0)	178 (75.1)	
Viral genotype			0.002			
1	635 (81.9)	300 (74.1)		124 (100)	237 (100)	
2 or 3	140 (18.1)	105 (25.9)		0	0	
AST (IU/L)			<0.001			0.038
<40	468 (58.9)	340 (73.9)		47 (37.6)	117 (49.4)	
≥40	327 (41.1)	120 (26.1)		78 (62.4)	120 (50.6)	
ALT (IU/L)			0.015			0.055
<40	509 (64.0)	338 (73.5)		39 (31.2)	99 (41.8)	
≥40	286 (36.0)	122 (26.5)		86 (68.8)	138 (58.2)	
HCV RNA (IU/L)						<0.001
<10^6^				33 (26.4)	113 (47.7)	
≥10^6^				92 (73.6)	124 (52.3)	
Platelets (10^9^/L)						0.039
<140				81 (64.8)	126 (53.2)	
≥140				44 (35.2)	111 (46.8)	
ALB (g/L)^b^				43.6 (40.8–46.2)	43.6 (40.9–46.2)	0.607
TP (g/L)^b^				78 (73.8–82.5)	78.1 (74.3–81.5)	0.920
WBC (10^9^/L)^b^				4.9 (3.9–6.1)	4.6 (3.7–5.5)	0.065
AFP (ng/mL) ^b^				3.8 (2.4–7.4)	3.3 (2.0–6.8)	0.160
HGB (g/L)^b^				139 (125–146)	133.5 (121–145)	0.056
GLU (mmol/L)^b^				5.5 (5.1–6.4)	5.6 (4.9–6.2)	0.531

Abbreviation: N-SVR, non sustained virological response; SVR, sustained virological response; Non-1b, genotype 2, 3; AST, aspartate transaminase; ALT, alanine aminotransferase; ALB, albumin; TP, total protein; WBC, white blood cell; AFP, alpha fetal protein; HGB, hemoglobin; GLU, glucose.

^a^Mean ± standard deviation.

^b^Median and interquartile range.

**Table 2:** Association of selected polymorphisms with HCV clearance and SVR.

Spontaneous clearance (n = 1255)					
Genotype	Chronic N(%)	Resolver N(%)	Clearance rate	OR (95% CI)^a^	*P* value^a^
rs12979860					
CC	634 (79.7)	404 (87.8)	38.9	1.00	
CT	134 (16.9)	51 (11.1)	27.6	0.63 (0.44–0.90)	0.010
TT	27 (3.4)	5 (1.1)	17.2	0.37 (0.14–0.99)	0.049
Dominant				0.59 (0.42–0.83)	0.002
Recessive				0.40 (0.15–1.06)	0.066
Additive				0.62 (0.46–0.83)	0.002
ss469415590					
TT/TT	628 (79.0)	409 (88.9)	39.4	1.00	
TT/ΔG	154 (19.4)	48 (10.4)	23.8	0.51 (0.36–0.73)	<0.001
ΔG/ΔG	13 (1.6)	3 (0.7)	18.7	0.37 (0.11–1.34)	0.130
Dominant				0.50 (0.36–0.71)	<0.001
Recessive				0.41 (0.12–1.47)	0.172
Additive				0.53 (0.39–0.74)	<0.001
**Response to treatment (n = 362)**					
**Genotype**	**N-SVR N(%)**	**SVR N(%)**	**SVR rate**	**OR (95% CI)^b^**	***P* value^b^**
rs12979860					
CC	65 (52.0)	180 (76.0)	73.5	1.00	
CT	37 (29.6)	42 (17.7)	53.2	0.41 (0.24–0.69)	<0.001
TT	23 (18.4)	15 (6.3)	39.5	0.24 (0.12–0.48)	<0.001
Dominant				0.34 (0.22–0.54)	<0.001
Recessive				0.30 (0.15–0.60)	0.001
Additive				0.46 (0.34–0.64)	<0.001
ss469415590					
TT/TT	91 (72.8)	212 (89.5)	70.0	1.00	
TT/ΔG	31 (24.8)	24 (10.1)	43.6	0.33 (0.18–0.60)	<0.001
ΔG/ΔG	3 (2.4)	1 (0.4)	25.0	0.14 (0.02–1.39)	0.094
Dominant				0.32 (0.18–0.56)	<0.001
Recessive				0.17 (0.02–1.67)	0.130
Additive				0.34 (0.20–0.58)	<0.001

Abbreviation: N-SVR, non sustained virological response; SVR, sustained virological response.

^a^Logistic regression analyses adjusted for age, gender, and viral genotype.

^b^Logistic regression analyses adjusted for age, gender, HCV RNA and platelets.

**Table 3:** Cumulative effects of rs12979860 and ss469415590 on HCV clearance and SVR.

Variables^a^	Spontaneous clearance			Response to treatment		
	Chronic N(%)	Resolver N(%)	OR (95% CI)^b^	N-SVR N(%)	SVR N(%)	OR (95% CI)^c^
0	572 (71.9)	385 (83.7)	1.00	61 (48.8)	174 (73.5)	1.00
1	111 (14.0)	39 (8.5)	0.52 (0.35–0.77)	16 (12.8)	34 (14.3)	0.81 (0.39–1.65)
2	82 (10.3)	34 (7.4)	0.62 (0.40–0.94)	42 (33.6)	23 (9.7)	0.13 (0.07–0.25)
3–4	30 (3.8)	2 (0.4)	0.10 (0.02–0.42)	6 (4.8)	6 (2.5)	0.18 (0.05–0.65)
Trend			*P*^d^ < 0.001			*P*^d^ < 0.001
0	572 (71.9)	385 (83.7)	1.00	61 (48.8)	174 (73.5)	1.00
1–4	223 (28.1)	75 (16.3)	0.50 (0.37–0.67)	64 (51.2)	63 (26.5)	0.28 (0.17–0.46)

Abbreviation: N-SVR, non sustained virological response; SVR, sustained virological response.

^a^Variables are numbers of combined unfavorable genotypes (rs12979860 T and ss469415590 ΔG).

^b^Logistic regression analyses adjusted for age, gender, and viral genotype.

^c^Logistic regression analyses adjusted for age, gender, HCV RNA and platelets.

^d^*P* value of Cochran-Armitage’s trend test.

**Table 4:** Multivariate stepwise regression analysis for independent factors of SVR.

Variables	Coef.	SE	OR (95% CI)	*P* value
rs12979860	−0.133	0.041	0.43 (0.25–0.75)	0.003
HCV RNA	−0.249	0.051	1.65 (1.02–2.67)	0.043
ss469415590	−0.222	0.067	0.29 (0.13–0.60)	<0.001
Platelets	1.870	0.049	0.28 (0.15–0.45)	<0.001

Abbreviation: Coef, coefficient of variation; SE, standard error; SVR, sustained virological response.

## References

[b1] Alter M. J. . Recommendations for prevention and control of hepatitis C virus (HCV) infection and HCV-related chronic disease. MMWR Morb Mortal Wkly Rep 47 (1998).

[b2] Lavanchy D. Evolving epidemiology of hepatitis C virus. Clinical Microbiology and Infection 17, 107–115 (2011). 21091831 10.1111/j.1469-0691.2010.03432.x

[b3] Seeff L. B. Natural history of chronic hepatitis C. Hepatology 36, S35–S46 (2002). 12407575 10.1053/jhep.2002.36806

[b4] Zeuzem S. . Expert opinion on the treatment of patients with chronic hepatitis C. Journal of viral hepatitis 16, 75–90 (2009). 18761607 10.1111/j.1365-2893.2008.01012.x PMC2759987

[b5] Huang C.-F. . Interleukin-28B genetic variants in identification of hepatitis C virus genotype 1 patients responding to 24 weeks peginterferon/ribavirin. Journal of hepatology 56, 34–40 (2012). 21703176 10.1016/j.jhep.2011.03.029

[b6] Tanaka Y. . Genome-wide association of IL28B with response to pegylated interferon-α and ribavirin therapy for chronic hepatitis C. Nature genetics 41, 1105–1109 (2009). 19749757 10.1038/ng.449

[b7] Prokunina-Olsson L. . A variant upstream of IFNL3 (IL28B) creating a new interferon gene IFNL4 is associated with impaired clearance of hepatitis C virus. Nature genetics 45, 164–171 (2013). 23291588 10.1038/ng.2521 PMC3793390

[b8] Bibert S. . IL28B expression depends on a novel TT/-G polymorphism which improves HCV clearance prediction. The Journal of experimental medicine 210, 1109–1116 (2013). 23712427 10.1084/jem.20130012 PMC3674704

[b9] Booth D. & George J. Loss of function of the new interferon IFN-[lambda] 4 may confer protection from hepatitis C. Nature genetics 45, 119–120 (2013). 23358218 10.1038/ng.2537

[b10] Urban T., Charlton M. R. & Goldstein D. B. Introduction to the genetics and biology of interleukin‐28B. Hepatology 56, 361–366 (2012). 22511448 10.1002/hep.25794

[b11] Nozawa Y. . Genetic polymorphism in IFNL4 and response to pegylated interferon‐α and ribavirin in Japanese chronic hepatitis C patients. Tissue antigens 83, 45–48 (2014). 24355007 10.1111/tan.12264

[b12] Covolo L. . The novel ss469415590 variant predicts virological response to therapy in patients with chronic hepatitis C virus type 1 infection. Alimentary pharmacology & therapeutics 39, 322–330 (2014). 24308755 10.1111/apt.12568

[b13] Knapp S. . Influence of IFNL3. rs12979860 and IFNL4. ss469415590 polymorphism on clearance of hepatitis C virus infection among Egyptians. Hepatology international 9, 251–257 (2015). 25788203 10.1007/s12072-015-9619-z

[b14] Stättermayer A. . Polymorphisms of interferon‐λ4 and IL28B–effects on treatment response to interferon/ribavirin in patients with chronic hepatitis C. Alimentary pharmacology & therapeutics 39, 104–111 (2014). 24205831 10.1111/apt.12547

[b15] Palmieri O. . Variation in genes encoding for interferon λ‐3 and λ‐4 in the prediction of HCV‐1 treatment‐induced viral clearance. Liver International 34, 1369–1377 (2014). 25283962 10.1111/liv.12411

[b16] Nagaoki Y. . Interferon lambda 4 polymorphism affects on outcome of telaprevir, pegylated interferon and ribavirin combination therapy for chronic hepatitis C. Hepatology Research 44, E447–E454 (2014). 24690180 10.1111/hepr.12336

[b17] Miyamura T. . IFNL4 ss469415590 Variant Is Associated with Treatment Response in Japanese HCV Genotype 1 Infected Individuals Treated with IFN-Including Regimens. International journal of hepatology 2014 (2014). 10.1155/2014/723868 PMC4274707 25548683

[b18] Real L. M. . IFNL4 ss469415590 variant shows similar performance to rs12979860 as predictor of response to treatment against hepatitis C virus genotype 1 or 4 in Caucasians. PloS one 9, e95515 (2014). 24748394 10.1371/journal.pone.0095515 PMC3991683

[b19] Honda M. . Hepatic ISG expression is associated with genetic variation in interleukin 28B and the outcome of IFN therapy for chronic hepatitis C. Gastroenterology 139, 499–509 (2010). 20434452 10.1053/j.gastro.2010.04.049

[b20] McGilvray I. . Hepatic Cell–Type Specific Gene Expression Better Predicts HCV Treatment Outcome Than IL28B Genotype. Gastroenterology 142, 1122–1131. e1121 (2012). 22285807 10.1053/j.gastro.2012.01.028

[b21] Sulkowski M. S. . Daclatasvir plus sofosbuvir for previously treated or untreated chronic HCV infection. New England Journal of Medicine 370, 211–221 (2014). 24428467 10.1056/NEJMoa1306218

[b22] Lange C. M. & Zeuzem S. Perspectives and challenges of interferon-free therapy for chronic hepatitis C. Journal of hepatology 58, 583–592 (2013). 23104162 10.1016/j.jhep.2012.10.019

[b23] Huang P. . Association of Polymorphisms in HLA Antigen Presentation-Related Genes with the Outcomes of HCV Infection. PloS one 10 (2015). 10.1371/journal.pone.0123513 PMC4395248 25874709

[b24] Huang P. . Genetic variants in antigen presentation-related genes influence susceptibility to hepatitis C virus and viral clearance: a case control study. BMC infectious diseases 14, 3837 (2014). 10.1186/s12879-014-0716-8 PMC4279674 25528575

[b25] Cui Q. . The association between the genetic polymorphisms of LMP2/LMP7 and the outcomes of HCV infection among drug users. Journal of biomedical research 24, 374–380 (2010). 23554652 10.1016/S1674-8301(10)60050-4 PMC3596683

[b26] Breiman L. Statistical modeling: The two cultures (with comments and a rejoinder by the author). Statistical Science 16, 199–231 (2001).

