# Supplementary material for: Genetic variants in interferon-λ 4 influences HCV clearance in Chinese Han population
Source: Sci Rep. 2017 Feb 10;7:42408. doi: 10.1038/srep42408 (PMC5301237; doi:10.1038/srep42408)
Supplement: Supplementary Information [file srep42408-s1.pdf]

# Genetic variants in interferon-λ 4 influences HCV clearance in Chinese Han population

Peng Huang[1], Yinan Yao[1], Ming Yue[2], Ting Tian[1], Hongbo Chen[3], Mingzhu Chen[1], Jie Wang[4], Yun Zhang[5], Rongbin Yu[1*]

**Supplemental Table 1. Information of primers and probes for TaqMan allelic discrimination**

| Polymorphism | | Sequence(5'-3') |
|---|---|---|
| *IL28B* rs12979860 | Primer | F: GCGCGGAGTGCAATTCAAC<br>R: TGTACTGAACCAGGGAGCTC |
| | Probe | FAM-CTGGTTCACGCCTTC-MGB<br>HEX-TGGTTCGCGCCTTC-MGB |
| *IFNL4* ss469415590 | Primer | F: GATGCGGCCGAGTGTCT<br>R: CTCCAGCGAGCGGTAGTG |
| | Probe | FAM-ATCGCAGCGGCCC-MGB<br>HEX-TCGCAGAAGGCCC-MGB |

**Supplemental Table 2. Association of selected polymorphisms with RVR and EVR**

| Genotype | N-RVR N(%) | RVR N(%) | OR (95% CI) | *P* value | N-EVR N(%) | EVR N(%) | OR (95% CI) | *P* value |
|---|---|---|---|---|---|---|---|---|
| rs12979860 | | | | | | | | |
| CC | 129 (59.4) | 116 (80.0) | 1.00 | | 37 (52.9) | 204 (69.9) | 1.00 | |
| CT | 67 (30.9) | 12 (8.3) | 0.19 (0.09-0.38) | < 0.001 | 29 (41.4) | 52 (17.8) | 0.32 (0.17-0.58) | < 0.001 |
| TT | 21 (9.7) | 17 (11.7) | 0.98 (0.49-1.97) | 0.951 | 4 (5.7) | 36 (12.3) | 1.41 (0.47-4.25) | 0.545 |
| Dominant | | | 0.37 (0.23-0.62) | < 0.001 | | | 0.46 (0.26-0.81) | 0.007 |
| Recessive | | | 1.36 (0.68-2.71) | 0.381 | | | 2.02 (0.69-5.96) | 0.199 |
| Additive | | | 0.67 (0.48-0.94) | 0.022 | | | 0.78 (0.53-1.15) | 0.206 |
| ss469415590 | | | | | | | | |
| TT/TT | 169 (77.9) | 134 (92.4) | 1.00 | | 38 (54.2) | 265 (90.8) | 1.00 | |
| TT/ΔG | 45 (20.7) | 10 (6.9) | 0.24 (0.11-0.53) | < 0.001 | 30 (42.9) | 25 (8.6) | 0.11 (0.05-0.23) | < 0.001 |
| ΔG/ΔG | 3 (1.4) | 1 (0.7) | 0.48 (0.05-4.78) | 0.528 | 2 (2.9) | 2 (0.7) | 0.12 (0.02-0.94) | 0.043 |
| Dominant | | | 0.26 (0.12-0.54) | < 0.001 | | | 0.11 (0.06-0.22) | < 0.001 |
| Recessive | | | 0.58 (0.06-5.71) | 0.638 | | | 0.21 (0.03-1.58) | 0.130 |
| Additive | | | 0.30 (0.15-0.60) | 0.001 | | | 0.15 (0.08-0.29) | < 0.001 |

Abbreviation: RVR, rapid virological response; EVR, early virological response.
Logistic regression analyses adjusted for age, gender, HCV RNA and platelets.

**Supplemental Table 3. Characteristics of association studies on *IFNL4* ss469415590 and HCV clearance and response to treatment**

**a) Studies on HCV spontaneous clearance**

| First author, date | Genotype/Model | Chronic/Resolver | OR (95% CI) | *P* value | Participants |
|---|---|---|---|---|---|
| Prokunina-Olsson et al. 2013 (UHS) | TT/TT | 45/30 | 3.51 | $7.9\times10^{-5}$ | African-American and European-American |
| | TT/ΔG | 168/53 | 1.66 | 0.056 | |
| | ΔG/ΔG | 137/26 | 1.00 | | |
| Prokunina-Olsson et al. 2013 (ALIVE) | TT/TT | 66/23 | 4.68 | $5.5\times10^{-6}$ | African-American |
| | TT/ΔG | 265/36 | 1.82 | 0.043 | |
| | ΔG/ΔG | 255/19 | 1.00 | | |
| Bibert et al. 2013 | TT/TT | 189/63 | | | White ethnicity |
| | TT/ΔG | 292/29 | | | |
| | ΔG/ΔG | 59/1 | | | |
| | Additive | | 0.28 (0.18-0.43) | $8.68\times10^{-9}$ | |
| Knapp et al. 2015 | TT/TT | 22/38 | 1.00 | | Egyptians |
| | TT/ΔG+ΔG/ΔG | 86/39 | 3.81 (1.99-7.28) | < 0.001 | |

**b) Studies on HCV response to treatment**

| First author, date | Genotype/Model | N-SVR/SVR | OR (95% CI) | *P* value | Participants |
|---|---|---|---|---|---|
| Prokunina-Olsson et al. 2013 (Virahep-C) | TT/TT | 9/7 | 2.90 | 0.067 | African-American and European-American |
| | TT/ΔG | 56/26 | 1.73 | 0.14 | |
| | ΔG/ΔG | 56/15 | 1.00 | | |
| Prokunina-Olsson et al. 2013 (HALT-C) | TT/TT | 6/2 | 11.0 | 0.027 | African-American and European-American |
| | TT/ΔG | 60/6 | 3.19 | 0.16 | |
| | ΔG/ΔG | 66/2 | 1.00 | | |
| Bibert et al. 2013 | TT/TT | 37/152 | | | White ethnicity |
| | TT/ΔG | 131/161 | | | |
| | ΔG/ΔG | 35/24 | | | |
| | Additive | | 0.38 (0.28-0.51) | $2.51\times10^{-10}$ | |
| Stättermayer et al. 2014 (Genotype 1) | TT/TT | 32/114 | | | Caucasians (98.8%) |
| | TT/ΔG | 119/107 | | | |

| | | | | | |
|---|---|---|---|---|---|
| | ΔG/ΔG | 31/29 | | | |
| | Additive | | 2.22 (1.63-3.02) | < 0.001 | |
| Stättermayer et al. 2014 (Genotype 2/3) | TT/TT | 17/70 | | | Caucasians (98.8%) |
| | TT/ΔG | 22/76 | | | |
| | ΔG/ΔG | 9/14 | | | |
| | Additive | | 1.51 (0.93-2.46) | 0.093 | |
| Stättermayer et al. 2014 (Genotype 4) | TT/TT | 5/32 | | | Caucasians (98.8%) |
| | TT/ΔG | 33/30 | | | |
| | ΔG/ΔG | 1/3 | | | |
| | Additive | | 5.36 (2.46-11.68) | < 0.001 | |
| Covolo et al. 2014 (Genotype 1) | TT/TT | 50/35 | 7.25 (1.91-27.51) | 0.004 | Caucasians |
| | TT/ΔG | 39/21 | 0.58 (0.19-1.83) | 0.36 | |
| | ΔG/ΔG | 10/9 | 1.00 | | |
| Covolo et al. 2014 (Genotype 2/3) | TT/TT | 5/59 | 1.60 (0.5-5.0) | 0.4 | Caucasians |
| | TT/ΔG+ΔG/ΔG | 4/85 | 1.00 | | |
| Nozawa et al. 2014 (Genotype 1) | TT/TT | 20/122 | 1.00 | | Japanese |
| | TT/ΔG+ΔG/ΔG | 32/40 | 4.73 (2.43-9.20) | 0.019 | |
| Miyamura et al. 2014 (Genotype 1) | TT/TT | 33/60 | | | Japanese |
| | TT/ΔG+ΔG/ΔG | 38/10 | 2.56 (1.01-6.56) | 0.049 | |
| Palmieri et al. 2014 (Genotype 1) | TT/TT | 36/74 | 4.73 (3.02-7.40) | $9.87\times10^{-13}$ | Caucasians |
| | TT/ΔG | 229/102 | | | |
| | ΔG/ΔG | 70/28 | | | |
| Nagaoki et al. 2014 (Genotype 1) | TT/TT | 13/163 | | < 0.001 | Japanese |
| | TT/ΔG+ΔG/ΔG | 44/63 | | | |

Abbreviation: N-SVR, non sustained virological response; SVR, sustained virological response.

Additive: TT/TT vs TT/ΔG vs ΔG/ΔG
